# 

**Published:** 1894-08

**Authors:** 


### CONTENTS—AUGUST, 1894.—VOL. X., NO. 2.

ORIGINAL COMMUNICATIONS—

The Treatment of Pulmonary Consumption. By Prof. Allen J. Smith, M. D., Galveston . . . . . . . . . . . . . . . . . . . . . . 49
Chloroform Administration. By Prof. Wm. Keiller, M. D., Galveston . 60
The Importance of Early Treatment in Nasal Obstructions By R. E. Moss, M. D . . . . . . . . . . . . . . . . . . . . . . . . . 70
Blindesss in Texas. H. L. Hillgartner, M. D., Austin. . . . . . . . . . 74

ABSTRACTS OF CURRENT MEDICAL LITERATURE—

Notes on Dermatology. Isadore Dyer, M. D . . . . . . . . . . . . . 78
Abstracts. By A. H. Goelet, M. D . . . . . . . . . . . . . . . . . 80

EDITORIAL—

Library and Museum of the Medical School. . . . . . . . . . . . . . 83
Obituary. . . . . . . . . . . . . . . . . . . . . . . . . . . . 84
Black Jaundice . . . . . . . . . . . . . . . . . . . . . . . . . 84
Prof. Smith's Paper . . . . . . . . . . . . . . . . . . . . . . . 85

MEDICAL NEWS AND MISCELLANY—

Many Interesting Paragraphs . . . . . . . . . . . . . . . . . . . 85

PUBLISHERS' NOTES . . . . . . . . . . . . . . . . . . . . . . . 88

